# Ultra-Processed Foods, MASLD, and Cognitive Aging: A Processing-Centered Gut–Liver–Brain Axis Perspective

**DOI:** 10.3390/nu18132041

**Published:** 2026-06-23

**Authors:** Yirui Chen, Hongxin Gui, Tieniu Zhao, Chang Liu, Ye Zhang, Mengyang Wang, Rongrong Yang

**Affiliations:** 1 School of Public Health, Tianjin University of Traditional Chinese Medicine, No. 10 Poyang Lake Road, Xiqing District, Tianjin 300193, China; ciandata@163.com (Y.C.); ghostcixi@163.com (H.G.); zhaotieniu@163.com (T.Z.); 2 Department of Exercise Biochemistry, School of Sports Science, Beijing Sport University, Beijing 100084, China; c.liu@bsu.edu.cn; 3 Centre for Epidemiology and Biostatistics, Melbourne School of Population and Global Health, University of Melbourne, Melbourne 3010, Australia; y.zhang@unimelb.edu.au; 4 Department of Neurobiology, Care Sciences and Society, Karolinska Institutet, Tomtebodavägen 18A Floor 10, 171 65 Solna, Sweden

**Keywords:** ultra-processed foods, MASLD, cognitive aging, dementia, gut–liver–brain axis, gut microbiome, metabolic endotoxemia, bile acids, food processing, neuroinflammation

## Abstract

**Background/Objectives:** Ultra-processed foods (UPFs) are increasingly recognized as dietary exposures associated with cardiometabolic, hepatic, and neurocognitive outcomes. However, UPFs are often treated mainly as nutrient-poor foods, whereas their processing-related features may perturb gut–liver–brain communication. This review examines whether metabolic dysfunction-associated steatotic liver disease (MASLD) can be conceptualized as a hepatic metabolic amplifier linking UPF exposure to cognitive aging. **Methods:** We conducted a structured narrative search of PubMed/MEDLINE, Web of Science Core Collection, and Scopus from January 2010 to 11 May 2026 across four evidence modules: UPFs and MASLD/NAFLD; UPFs and cognitive aging or dementia; UPFs and gut–liver–brain mechanisms; and MASLD/NAFLD and cognitive aging. Representative studies were prioritized according to direct relevance to the proposed axis, study design, exposure and outcome validity, mechanistic specificity, and contribution to major evidence gaps. **Results:** Observational and mechanistic evidence links higher UPF consumption with liver steatosis, MASLD/NAFLD-related outcomes, cognitive decline, cognitive impairment, stroke, and dementia-related outcomes, although causality remains incompletely established and residual confounding is important. Candidate pathways include food-matrix disruption, rapid eating, displacement of microbial substrates, selected additives and processing-derived compounds, intestinal barrier dysfunction, metabolic endotoxemia, bile acid signaling, hepatic lipotoxicity, systemic inflammation, vascular dysfunction, and neuroimmune activation. Many pathways overlap with general cardiometabolic dysfunction; the processing-centered contribution lies in positioning industrial formulation as an upstream exposure and MASLD as a hepatic node that may amplify gut-derived and metabolic signals relevant to brain aging. **Conclusions:** A processing-centered gut–liver–brain framework integrates UPFs, MASLD, and cognitive aging as linked metabolic-aging phenomena. Future studies should test UPF substitution using liver imaging, microbiome profiling, metabolomics, bile acid and inflammatory biomarkers, neuroimaging, and cognitive assessment.

## 1. Introduction

Metabolic dysfunction-associated steatotic liver disease (MASLD), formerly largely discussed under the term non-alcoholic fatty liver disease (NAFLD), has become one of the most common chronic liver conditions worldwide and is tightly linked to obesity, insulin resistance, dyslipidemia, type 2 diabetes, cardiovascular disease, and systemic inflammation [1,2]. Cognitive aging and dementia represent a parallel public health challenge in aging societies. Although liver disease and cognitive decline are often managed in different clinical silos, they share a set of upstream biological processes: metabolic dysfunction, chronic low-grade inflammation, endothelial injury, gut microbial dysbiosis, altered bile acid metabolism, and impaired insulin signaling.

The nomenclature shift from NAFLD to MASLD is not merely semantic. It makes explicit that steatotic liver disease commonly arises in a broader state of metabolic dysfunction and that extrahepatic outcomes are part of the disease context [1,3]. For cognitive aging research, this reframing is useful because the brain is strongly exposed to the same metabolic milieu that accompanies hepatic steatosis: insulin resistance, adipose inflammation, dyslipidemia, hypertension, and endothelial injury. Rather than asking only whether “fatty liver” is independently associated with dementia, the more informative question is how hepatic metabolic dysfunction participates in multi-organ aging.

Much nutritional research has focused on protective dietary patterns such as the Mediterranean diet, the Dietary Approaches to Stop Hypertension (DASH) diet, the Mediterranean-DASH Intervention for Neurodegenerative Delay (MIND) diet, and other plant-forward dietary models. A complementary exposure has moved rapidly into the center of public nutrition: ultra-processed food consumption. The NOVA classification defines UPFs according to the extent and purpose of industrial processing rather than only nutrient composition, a distinction that is central to this review because processing can alter food structure, matrix integrity, texture, eating rate, additive exposure, microbial substrate availability, and processing-derived compounds [4].

A 2024 umbrella review in *The BMJ* synthesized evidence across 45 pooled analyses including nearly 10 million participants and reported associations between UPF exposure and multiple adverse health outcomes [5]. Other umbrella reviews similarly conclude that the overall evidence is strongest for cardiometabolic outcomes, while evidence for some organ-specific outcomes remains less certain and heterogeneous [6]. For the present review, these umbrella findings provide public health context rather than a mechanistic explanation. If UPFs are associated with multiple outcomes, the key task is to identify which biological pathways are most plausible for liver–brain aging, which pathways mainly reflect poor diet quality or adiposity, and which remain speculative.

The public health relevance of UPFs extends beyond individual diet quality. UPFs are cheap, shelf-stable, aggressively marketed, and deeply embedded in modern food systems. They are also increasingly considered in relation to cardiometabolic disease, gastrointestinal disorders, including irritable bowel syndrome, mental health, cancer, and planetary health [7,8,9,10,11,12]. This breadth creates a risk of conceptual dilution: if UPFs are linked to almost everything, the task for a mechanistic review is to specify which pathways are plausible, which outcomes are most biologically coherent, and where the evidence remains weak.

The central problem is that UPFs are too often reduced to a shorthand for “high sugar, high fat, high salt, low fiber”. That description is partly true but conceptually incomplete. UPFs should also be understood as processing-related dietary exposures: industrial formulations in which the food matrix is disrupted, fiber and polyphenol-rich substrates are displaced, texture and eating rate are altered, additives may be introduced, and hyperpalatable combinations may promote passive overconsumption [13,14]. These processing features may be especially relevant to the gut–liver–brain axis because the intestine and liver are the first biological interfaces exposed to dietary structure, microbial substrates, additives, microbial metabolites, lipopolysaccharide (LPS), and bile acids.

Bile acid signaling is introduced early because it provides an intrinsically gut–liver–brain pathway. Gut microbes transform bile acids, the liver regulates bile acid synthesis and enterohepatic circulation, and bile acids signal through receptors such as farnesoid X receptor (FXR) and Takeda G protein-coupled receptor 5 (TGR5). Perturbation of this network may influence hepatic lipid metabolism, intestinal barrier integrity, inflammation, glucose regulation, and neuroimmune signaling.

Previous reviews have examined UPFs in relation to cardiometabolic disease, gut health, liver disease, or brain health separately. Few have integrated UPFs, MASLD, cognitive aging, and gut–liver–brain mechanisms into a unified processing-centered framework. This review advances the hypothesis that MASLD may act as a hepatic metabolic amplifier linking food processing to brain aging. In this framework, UPFs disrupt gut microbial ecology and intestinal barrier integrity, promote hepatic steatosis and inflammation, and amplify systemic metabolic, inflammatory, vascular, and neuroimmune signals that may accelerate cognitive aging.

The conceptual novelty of this review is therefore not the claim that UPFs are “unhealthy foods”. Instead, we argue that food processing can be treated as an upstream exposure that reshapes the physical, chemical, microbial, behavioral, and social properties of the diet. MASLD is positioned as a biologically plausible hepatic node within this pathway: the liver receives gut-derived and diet-derived signals through portal circulation, converts them into inflammatory and metabolic outputs, and communicates with the brain through vascular, immune, endocrine, hepatokine, adipokine, and bile acid pathways. This storyline is summarized as: from food processing to metabolic brain aging, with MASLD as the missing hepatic link.

## 2. Methods: Search Strategy and Selection Criteria

This structured narrative review was designed to synthesize converging epidemiological and mechanistic evidence rather than to conduct a formal systematic review. We therefore did not perform duplicate screening, formal risk-of-bias scoring, or PRISMA flow-chart reporting. To improve transparency, we developed a structured search strategy organized around four evidence modules and used these searches to identify primary epidemiological studies, clinical studies, systematic reviews, meta-analyses, controlled feeding studies, mechanistic human studies, and selected preclinical studies with clear relevance to human gut–liver–brain pathways.

The search strategy was developed for PubMed/MEDLINE and adapted for Web of Science Core Collection and Scopus. Searches covered records from January 2010 to 11 May 2026, with earlier landmark mechanistic studies included when necessary to define core concepts. The four modules were: (1) UPFs and MASLD/NAFLD; (2) UPFs and cognitive aging, cognitive decline, or dementia; (3) UPFs and gut–liver–brain mechanisms; and (4) MASLD/NAFLD and cognitive aging. Search terms included variants of “ultra-processed food”, “NOVA classification”, “food processing”, “MASLD”, “NAFLD”, “hepatic steatosis”, “cognitive decline”, “dementia”, “gut microbiome”, “intestinal permeability”, “LPS”, “short-chain fatty acids”, “bile acids”, “FXR”, “TGR5”, “blood-brain barrier”, and “neuroinflammation”. Full search strings, including the complete Web of Science Core Collection strategy, are provided in Supplementary Table S1.

Eligible literature included prospective cohort studies, cross-sectional studies, randomized or controlled feeding studies, systematic reviews and meta-analyses, mechanistic studies in humans, and selected preclinical studies with direct relevance to the proposed pathways. Evidence was prioritized when it directly informed one of five questions: whether UPF intake is associated with liver steatosis or MASLD; whether UPF intake is associated with cognitive decline, dementia, stroke, or related brain-health outcomes; whether processing-related features influence the gut microbiome, barrier integrity, bile acid metabolism, inflammatory tone, or metabolic signaling; whether MASLD or fibrosis is associated with cognitive or structural brain outcomes; and whether replacement of UPFs with minimally processed foods is a plausible intervention target.

For the evidence tables, representative studies were selected using the following hierarchy: (i) systematic reviews or meta-analyses summarizing a defined evidence domain; (ii) prospective cohort studies with explicit NOVA-based exposure assessment or validated liver or cognitive outcomes; (iii) studies using imaging, elastography, omics, or biomarker-based outcomes; (iv) studies that distinguished UPF subtypes or used replacement models; and (v) mechanistic human or preclinical studies that clarified a pathway central to the proposed framework. Studies were not prioritized when they focused only on isolated nutrients without food-processing classification, pediatric-only populations without relevance to life-course interpretation, conference abstracts without full text, or overt cirrhosis or hepatic encephalopathy rather than MASLD-related cognitive aging, unless they were used narrowly as clinical context for gut–liver–brain communication.

Because much of the available evidence was generated before the nomenclature shift from NAFLD to MASLD, we retained the original terminology when describing study-specific definitions and outcomes. The term MASLD is used when studies explicitly applied current criteria or when discussing the contemporary conceptual framework of steatotic liver disease in the context of metabolic dysfunction. NAFLD-based evidence is therefore interpreted as overlapping but not identical to current MASLD evidence.

Because the aim was integrative rather than exhaustive, the evidence tables present selected representative studies and syntheses that anchor the main argument. They are intended to show convergence, heterogeneity, and methodological gaps across domains rather than to function as PRISMA-style complete extraction tables.

## 3. Ultra-Processed Foods as a Processing Exposure

### 3.1. NOVA Classification and Biological Relevance

The NOVA classification groups foods according to the extent and purpose of industrial processing, with UPFs defined as formulations of ingredients, often including substances derived from foods and additives, that are designed to be convenient, durable, palatable, and profitable [4]. NOVA is currently the dominant framework in epidemiological research on UPFs, but its scientific relevance depends on whether processing adds explanatory value beyond conventional nutrient profiling.

A key controlled feeding study suggests that it can. In a randomized inpatient crossover trial, Hall and colleagues provided adults with *ad libitum* ultra-processed and unprocessed diets for two weeks each. The menus were designed to be matched for presented calories, macronutrients, sugar, sodium, and fiber, yet participants consumed about 500 kcal/day more during the UPF diet and gained weight, whereas they lost weight during the unprocessed diet phase [13]. This trial does not isolate every processing attribute, but it demonstrates that industrially formulated diets can alter energy intake under highly controlled conditions even when nutrient targets appear similar.

The trial also highlights why UPFs should not be treated as interchangeable with single nutrients. Processing modifies texture, structure, sensory exposure, energy density, water content, eating rate, and satiety kinetics. A meal composed of UPFs may be consumed more rapidly and with lower satiation than a minimally processed meal, and eating rate itself is increasingly recognized as a behavioral pathway linking food structure to energy intake [15]. This behavioral dimension overlaps with work on food reward, craving, and food-addiction-like phenotypes, while endocrine and obesity reviews emphasize that hyperpalatable industrial formulations can sustain repeated energy surplus in vulnerable environments [16,17]. This matters for MASLD because sustained positive energy balance and visceral adiposity increase hepatic lipid flux, while it matters for cognition because obesity and insulin resistance contribute to vascular and neuroinflammatory brain injury.

### 3.2. Food Matrix Disruption, Additives, and Hyperpalatability

Processing may matter through several overlapping mechanisms. First, disruption of the food matrix can increase eating rate, reduce oral processing demands, alter satiety signaling, and accelerate digestion. Second, UPF-rich diets often displace intact fiber, resistant starch, and polyphenol-rich plant foods that provide substrates for short-chain fatty acid (SCFA)-producing microbes. Third, industrial formulations may contain emulsifiers, sweeteners, colorants, stabilizers, preservatives, and microparticles. The human evidence for individual additives remains uneven, but preclinical and emerging clinical literature indicates that selected additives can influence the gut microbiome, intestinal permeability, and intestinal inflammation [14,18,19]. Fourth, UPFs are frequently engineered for hyperpalatability, convenience, low cost, and rapid eating, all of which may promote sustained energy surplus and visceral adiposity.

The additive literature is especially important but requires a careful tone. Reviews of UPFs and gut health emphasize that epidemiological evidence for UPFs is largely observational, whereas mechanistic evidence for additives often comes from cell and animal models [14,20,21]. Recent narrative reviews also converge on the same methodological message: additives, matrix disruption, and gut microbial effects should be studied as related but separable features of industrially formulated foods rather than collapsed into a single exposure [22,23,24]. Some emulsifiers can perturb mucus-microbiota interactions and promote metabolic or inflammatory phenotypes in mice [18]; long-term exposure to Tween 80 has been linked with hepatic lipid accumulation and inflammatory changes in young and aged mice [25]; non-nutritive sweeteners can modify glucose tolerance and microbial ecology in selected experimental contexts [19]; and maternal emulsifier exposure has been shown in mice to program offspring metabolic and neurobehavioral outcomes [26]. These findings are not sufficient to assign equal risk to all additives, but they support a broader mechanistic principle: industrial formulation can alter host-microbe interactions independently of macronutrient composition.

UPFs also concentrate or co-occur with compounds that may be relevant to oxidative and vascular stress, including advanced glycation end products, nitrites in processed meats, oxidized lipids, and contaminants generated or concentrated during processing and high-temperature preparation [27,28]. These exposures are difficult to separate from food categories and cooking methods in observational studies, but they add plausibility to a processing-centered model that includes oxidative stress, endothelial dysfunction, and inflammatory tone. Table 1 summarizes the processing specificity of these candidate pathways and their gut, liver, and brain relevance.

### 3.3. Limitations and Controversies of UPF Classification

NOVA is useful but imperfect. UPFs are heterogeneous, and not all UPF subtypes are biologically equivalent. Sugar-sweetened beverages, processed meats, sweet bakery products, and savory snacks plausibly differ from fortified cereals, some whole-grain packaged products, and selected plant-based alternatives. UPF classification can also be difficult when dietary instruments lack brand-level or ingredient-level detail. Therefore, this review does not treat UPFs as a single toxic category. Instead, it uses UPF exposure as a marker of processing-related dietary stress while emphasizing subtype analyses, replacement models, and mechanistic biomarkers as essential next steps.

This nuance is important for both science and translation. Overly broad anti-UPF messaging can obscure differences among products and may be less useful for populations facing food insecurity, limited cooking time, or constrained access to fresh foods. Conversely, relying only on nutrient profiling can miss matrix disruption, speed of intake, additive exposure, and displacement of microbial substrates. A mature framework therefore requires both nutrient and processing dimensions, ideally combined with food-category, subtype, and replacement analyses [6,29,30].

## 4. Evidence Linking UPFs to MASLD

### 4.1. Epidemiological Evidence

The epidemiological evidence linking UPFs to fatty liver outcomes has grown quickly. Early systematic reviews found that higher UPF consumption was associated with NAFLD, metabolic syndrome, and insulin resistance, but also noted heterogeneity in study design, dietary assessment, and liver outcome ascertainment [31,32]. A systematic review and meta-analysis of adult studies reported that high versus low UPF intake was associated with higher NAFLD risk, with evidence suggestive of a dose-response pattern [32]. An updated systematic review and dose-response meta-analysis including 10 articles, 513,440 participants, and 20,637 NAFLD cases found that highest UPF consumption was associated with a 22% higher NAFLD risk and that each 10% increment in UPF consumption was associated with a 6% higher risk, although heterogeneity was substantial [33].

Prospective evidence is particularly important because cross-sectional studies cannot separate dietary antecedents from consequences of metabolic disease. In a UK Biobank cohort of 173,889 participants, higher UPF intake assessed by repeated 24 h dietary recalls and NOVA classification was associated with increased risk of NAFLD, liver fibrosis/cirrhosis, and severe liver disease during a median follow-up of 8.9 years [34]. A subsequent UK Biobank analysis linked metabolomic and proteomic signatures of UPF intake with MASLD and other adverse liver outcomes, implicating lipid metabolism, immune, and inflammatory pathways [35]. Cross-sectional elastography-based data from NHANES also support an association between higher UPF intake and controlled attenuation parameter values, suggesting greater liver steatosis, while associations with liver stiffness appear less consistent [36].

Additional population-based studies broaden this evidence base. Analyses using NHANES data reported positive associations between UPF intake and odds of NAFLD in adolescents and adults [37,38]. The PREDIMED-Plus cohort linked UPF consumption with NAFLD-related biomarkers in older adults with overweight or obesity and metabolic syndrome [39], while earlier clinical and population work connected UPF intake with metabolic syndrome features and NAFLD markers [40]. Prospective data from Asian cohorts suggest that UPF intake and selected UPF subtypes may predict incident NAFLD or MASLD, although subtype patterns vary by food culture and dietary assessment method [41,42,43]. Evidence from pediatric obesity populations further suggests that higher UPF intake may track with moderate-to-severe MASLD and insulin resistance, making life-course exposure an important concern [44,45]. Emerging regional studies from Korea, Greece, and Iran further indicate that the UPF-liver association is not confined to one dietary culture, although harmonized MASLD definitions and food-classification rules remain essential [46,47].

The subtype question is crucial. Some studies suggest stronger liver associations for sugar-sweetened beverages, instant noodles, processed meats, sweet snacks, or high-fat packaged products than for all UPFs combined [41,42]. This pattern fits hepatic biology: fructose-rich beverages can increase de novo lipogenesis; processed meats and high-temperature products can contribute nitrites, heme iron, oxidized lipids, and advanced glycation end products; and refined starch products can increase glycemic and insulin demand [27,48]. Yet subtype analyses remain inconsistent because food categories are not harmonized across cohorts.

Taken together, Table 2 shows that the UPF-MASLD literature is no longer limited to isolated cross-sectional findings. The evidence now includes systematic reviews, dose-response synthesis, prospective cohorts, national survey analyses, omics work, pediatric/adolescent studies, and geographically diverse cohorts. The most important remaining limitation is not whether a signal exists, but whether future studies can separate processing level from food category, nutrient profile, adiposity mediation, socioeconomic context, and MASLD diagnostic heterogeneity.

The most biologically plausible interpretation is layered. Some pathways are likely mediated by overall diet quality and adiposity, whereas others may be more specific to industrial processing, including food-matrix disruption, rapid eating, selected additives, processing-derived compounds, and displacement of microbial substrates. This distinction is important because a processing-centered framework does not deny the role of obesity or diet quality; rather, it asks whether ultra-processing adds explanatory value beyond these conventional pathways.

### 4.2. Potential Pathways to Hepatic Steatosis and Inflammation

Three pathway clusters are especially plausible. The first is energy surplus and visceral adiposity. UPFs can increase eating rate and passive overconsumption, facilitating weight gain, visceral adipose expansion, and ectopic lipid deposition [13]. The second is refined carbohydrate, added sugar, and fructose exposure. Sugar-sweetened beverages, refined starches, and sweet packaged products can increase hepatic substrate delivery, de novo lipogenesis, hypertriglyceridemia, and insulin resistance, all central to MASLD pathogenesis [2,48]. Fructose is a useful example because fructose-rich beverages can preferentially increase hepatic lipogenesis under conditions of energy surplus, although fructose should not be treated as the only relevant UPF component [49]. The third is gut-derived inflammation. Low-fiber UPF-rich diets may reduce SCFA production and impair intestinal barrier integrity, while selected additives, dysbiosis, and processing-derived compounds may increase metabolic endotoxemia. The liver, receiving portal blood directly from the intestine, is a first-line sensor of this inflammatory and microbial traffic.

These pathways are not mutually exclusive. UPFs may simultaneously increase total energy intake, reduce protective food components, alter microbial metabolism, increase repeated glycemic and lipogenic exposure, and increase exposure to additives or processing-derived compounds. Lipotoxic intermediates, mitochondrial stress, Kupffer cell activation, and hepatic stellate-cell activation can then connect steatosis with inflammation and fibrogenesis. Reviews of diet and MASLD increasingly emphasize that inappropriate dietary patterns promote hepatic de novo lipogenesis, insulin resistance, gut–liver dysfunction, inflammation, and oxidative stress [2,3,50]. The most biologically plausible model is therefore multi-hit: food processing increases the probability that several hepatic stressors converge in the same individual.

### 4.3. Evidence Gaps

Several limitations prevent strong causal inference. Many studies still use NAFLD terminology and do not fully operationalize current MASLD criteria. Liver outcomes vary widely, including fatty liver index, ultrasound, controlled attenuation parameter, liver enzymes, diagnostic codes, MRI-based fat measures, and fibrosis scores. Dietary assessment usually relies on FFQs or 24 h recalls, often without complete ingredient or brand information. Residual confounding remains possible because UPF intake clusters with socioeconomic status, sleep, physical activity, smoking, depression, and healthcare access. Still, the convergence of prospective, meta-analytic, elastography, biomarker, and omics evidence makes UPF exposure a credible target for MASLD prevention research.

Another gap is mediation. Adiposity attenuates many UPF-liver associations, but this should not be interpreted as simple confounding. Adiposity may be a mediator through which UPF exposure contributes to hepatic lipid accumulation, while gut permeability, bile acids, inflammatory proteins, triglyceride-rich lipoproteins, and insulin resistance may represent parallel mediating pathways. Future cohorts should therefore estimate direct, indirect, and replacement effects instead of treating body mass index adjustment as a final answer.

## 5. MASLD as a Hepatic Metabolic Amplifier

### 5.1. The Liver as a Portal Sensor of Processing-Related Dietary Stress

The liver occupies a strategic position in the gut–liver–brain axis. It receives portal venous blood enriched with dietary carbohydrates and lipids, microbial metabolites, bile acids, LPS, and inflammatory signals from the intestine. A UPF-rich dietary pattern may therefore first appear as hepatic metabolic stress: increased de novo lipogenesis, impaired insulin signaling, altered lipid export, mitochondrial stress, Kupffer cell activation, and hepatocellular injury. Once MASLD develops, the liver may no longer be a passive target. It may become an amplifier of the original dietary and gut-derived signals.

**Figure 1:**
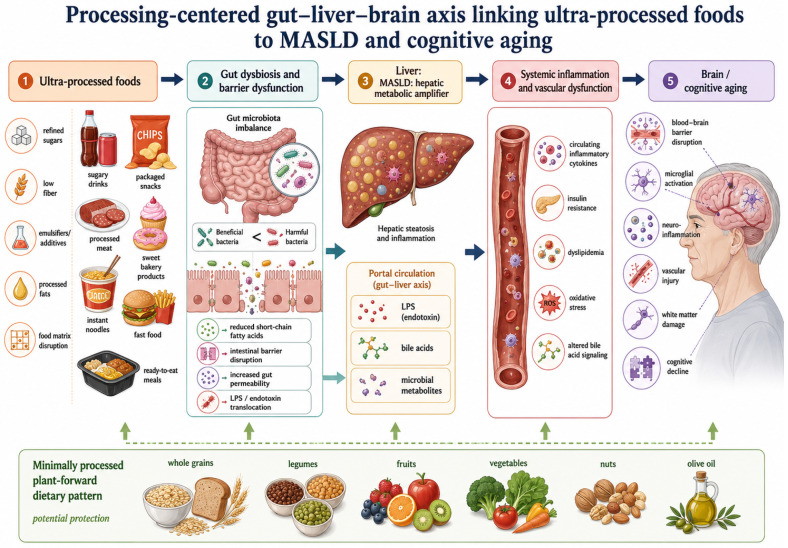
Processing-centered gut–liver–brain framework linking ultra-processed foods (UPFs) to metabolic dysfunction-associated steatotic liver disease (MASLD) and cognitive aging. UPF features may disrupt the gut microbiome and intestinal barrier, increase lipopolysaccharide (LPS) translocation and bile acid perturbation, promote hepatic steatosis and inflammation, and amplify systemic inflammatory, vascular, metabolic, and neuroimmune signals relevant to metabolic brain aging. MASLD is positioned as a hepatic metabolic amplifier rather than merely a parallel diet-related outcome. The figure was assembled in Microsoft PowerPoint and exported as PNG.

We propose the following central model: MASLD serves as a hepatic relay and amplifier that converts processing-related dietary stress into systemic metabolic and neuroinflammatory signals. As shown in Figure 1, this framework helps connect findings that otherwise appear dispersed across fields: UPF-rich diets are linked to steatosis and inflammatory biomarkers; MASLD and liver fibrosis are linked to brain structure and cognitive outcomes; and gut microbial, barrier, bile acid, and inflammatory pathways bridge the two.

Several features make the liver a plausible amplifier rather than a passive bystander. Hepatocytes respond to carbohydrate and lipid oversupply by increasing de novo lipogenesis, very-low-density lipoprotein handling, oxidative metabolism, and stress signaling. Kupffer cells and recruited macrophages respond to gut-derived microbial products through innate immune pathways. Hepatic stellate cells respond to inflammatory and lipotoxic injury with fibrogenic activation. Once this hepatic inflammatory state is established, circulating cytokines, acute-phase proteins, altered lipoproteins, hepatokines, bile acids, and coagulation-related mediators may influence the vascular and neuroimmune environment. This is the biological reason MASLD may help translate a dietary exposure into a systemic aging phenotype.

### 5.2. MASLD-Mediated Systemic Inflammation and Vascular Dysfunction

MASLD is a systemic metabolic disease rather than a liver-limited phenotype. It is associated with chronic inflammation, insulin resistance, dyslipidemia, endothelial dysfunction, and cardiovascular disease [2]. These same processes contribute to cerebral small-vessel disease, white matter injury, stroke, blood–brain barrier (BBB) dysfunction, and executive dysfunction. In this sense, MASLD may mark a state in which the liver magnifies the consequences of diet-induced gut and metabolic stress.

Evidence linking NAFLD/MASLD to brain outcomes is mixed but biologically suggestive. In a cross-cohort collaboration pooling middle- and older-age adults from the Framingham Heart Study, Rotterdam Study, and Study of Health in Pomerania, NAFLD was associated with smaller total brain and gray matter volumes, and liver fibrosis was associated with smaller total brain volume [51]. In a prospective cross-sectional clinical study, cognitive deficits were common in individuals with NAFLD, and liver fibrosis was modestly associated with worse inhibitory control and attention [52]. Meta-analytic evidence has linked NAFLD with cognitive impairment, although dementia subtype results are inconsistent [53,54,55]. A 2025 meta-analysis focused on liver fibrosis reported a 32% higher long-term risk of incident dementia, with stronger associations at more advanced fibrosis stages and for vascular dementia than Alzheimer disease [56]. Mendelian-randomization and observational analyses have also begun to test whether genetically proxied or clinically defined NAFLD relates to dementia and brain phenotypes, but these approaches remain sensitive to instrument choice, pleiotropy, and phenotype definition [57,58].

Large-scale population studies add depth and caution. UK Biobank analyses have associated liver dysfunction, fibrosis/cirrhosis, and sensitivity analyses for MASLD with incident dementia, cognition, and brain structural changes [59]. Other cohorts suggest that liver integrity markers may predict Alzheimer disease and related dementias [60], and UK Biobank neuroimaging has been used to characterize brain morphology associated with MASLD [61]. Earlier work also linked NAFLD to poorer cognitive test performance, supporting a continuum from metabolic liver dysfunction to measurable cognitive vulnerability before dementia diagnosis [62,63]. Reviews of NAFLD and neurodegeneration similarly emphasize shared inflammatory, metabolic, and vascular mechanisms while noting that causality remains unresolved [64]. At the same time, some studies have reported null or inverse associations between NAFLD/MASLD and incident dementia after adjustment [54,65,66]. These inconsistencies likely reflect differences in age, survival bias, diagnostic definitions, fibrosis burden, competing cardiometabolic risks, medication exposure, and whether the study captures steatosis alone or advanced fibrotic disease.

The distinction between steatosis and fibrosis may be central. Simple steatosis can be metabolically heterogeneous, whereas fibrosis is a stronger marker of chronic hepatic injury and systemic disease burden. This may explain why fibrosis-focused evidence appears more consistently linked to dementia and brain aging than NAFLD/MASLD defined by fatty liver index or administrative codes alone [52,56]. For the UPF framework, fibrosis may represent a later and more amplified stage of the same processing-metabolic pathway.

### 5.3. MASLD as a Bridge Between Gut Dysbiosis and Brain Aging

The gut–brain axis can operate without MASLD, but MASLD may intensify gut-derived signals by placing the liver in an inflamed, insulin-resistant, and lipotoxic state. Portal endotoxin exposure may activate Kupffer cells and hepatic stellate cells; hepatic inflammation can increase circulating cytokines and acute-phase reactants; altered bile acid metabolism can reshape gut microbial ecology and host metabolic signaling; and dyslipidemia can impair endothelial function. These processes plausibly increase vulnerability to BBB dysfunction, microglial activation, and vascular brain injury. The key conceptual move is therefore to treat MASLD not only as another outcome of poor diet, but as an intermediate amplifier in the pathway from food processing to metabolic brain aging.

Preclinical evidence supports the plausibility of this bridge. Experimental MASLD models have shown cognitive dysfunction associated with systemic inflammation and neuroinflammation [67]; diet-induced NAFLD models have linked hepatic metabolic injury with brain dysfunction [68]; and NAFLD can exacerbate environmental or inflammatory second hits that promote BBB dysfunction and NLRP3-dependent neuroinflammation [69]. Although animal models cannot reproduce the complexity of human UPF exposure, they are useful for testing portal endotoxin, bile acid, inflammatory, and BBB mechanisms that are difficult to isolate in cohorts.

Clinical microbiota-directed intervention studies provide supportive context, but they should not be overinterpreted as direct UPF-MASLD evidence. For example, a prospective clinical trial of fecal microbiota transplantation in alcohol-associated cirrhosis reported short-term improvements in hepatic encephalopathy severity, liver stiffness, steatosis measures, and inflammatory markers [70]. Although this was a cirrhosis and hepatic encephalopathy setting rather than MASLD or cognitive aging, it supports the broader clinical relevance and potential modifiability of gut–liver signaling in chronic liver disease.

### 5.4. Bile Acid-FXR-TGR5 Signaling

Bile acids are not only digestive detergents but also metabolic signaling molecules acting through farnesoid X receptor (FXR), Takeda G protein-coupled receptor 5 (TGR5), and other pathways. Gut microbes modify primary into secondary bile acids, while bile acids reciprocally shape microbial ecology. UPF-related dysbiosis may perturb bile acid pools, potentially influencing hepatic lipid metabolism, glucose regulation, intestinal barrier function, inflammation, and brain metabolic signaling. This bile acid pathway is a plausible liver–brain bridge but remains under-tested in UPF intervention studies.

The bile acid pathway is particularly appealing for this review because it is intrinsically gut–liver–brain. Bile acid profiles can serve as biomarkers of metabolic disease and may reflect interactions among hepatic synthesis, microbial transformation, intestinal absorption, and receptor signaling [71]. In experimental NAFLD/NASH, altered circulating bile acids have been linked with changes in brain FXR/TGR5 expression, BBB markers, and microglial activation [72]. Human UPF studies rarely measure bile acids, which leaves an important mechanistic gap.

### 5.5. Adipokine Signaling and Adipose–Liver–Brain Crosstalk

Adipose-derived endocrine signaling is another amplifier that should be separated from processing-specific mechanisms. UPF-rich diets may contribute to positive energy balance and visceral adiposity, but leptin resistance, reduced adiponectin, resistin signaling, free fatty acid flux, and inflammatory adipokines are not unique to UPFs. They are broader cardiometabolic mediators that can intensify hepatic insulin resistance, steatosis, endothelial dysfunction, and neuroinflammatory vulnerability [73].

This distinction strengthens rather than weakens the proposed framework. Processing-related features may initiate or sustain excess intake and gut stress, while adipokine disruption can amplify the consequences once visceral adiposity and MASLD are established. In this sense, adipokine signaling represents an adiposity-mediated amplification pathway linking obesity, MASLD, vascular dysfunction, and cognitive aging, not a mechanism that should be described as specific to ultra-processing.

## 6. Mechanistic Pathways Across the Gut–Liver–Brain Axis

**Figure 2:**
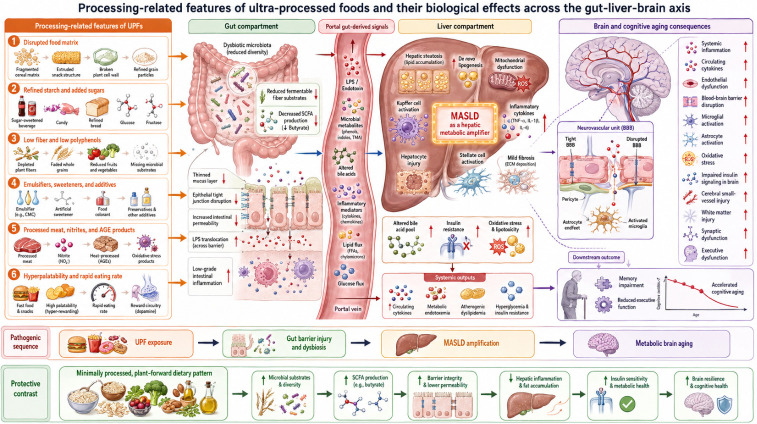
Processing-related features of ultra-processed foods (UPFs) and their candidate biological effects across the gut–liver–brain axis. Different UPF features may engage overlapping mechanisms, including altered eating rate, reduced microbial substrate availability, short-chain fatty acid (SCFA) depletion, gut barrier injury, lipopolysaccharide (LPS) translocation, hepatic de novo lipogenesis (DNL), mitochondrial stress, Kupffer cell activation, bile acid pool alteration, insulin resistance, systemic inflammation, blood–brain barrier (BBB) dysfunction, microglial activation, endothelial dysfunction, small-vessel injury, and executive dysfunction. The protective contrast highlights how minimally processed, plant-forward dietary patterns may support microbial substrates, SCFA production, barrier integrity, hepatic metabolic health, and brain resilience. The figure was assembled in Microsoft PowerPoint and exported as PNG.

Mechanistic evidence is strongest when viewed as a network rather than as isolated pathways. UPF exposure can simultaneously alter food structure, substrate availability, additive exposure, eating behavior, glycemic load, lipid flux, and the intestinal microbial ecosystem. These upstream changes may converge on gut barrier injury, MASLD, systemic inflammation, vascular dysfunction, and brain aging (Figure 2).

The mechanistic framework should be read in two layers. Gut dysbiosis, endotoxemia, insulin resistance, systemic inflammation, vascular dysfunction, and neuroimmune activation are established components of cardiometabolic and gut–liver–brain research. The more specific contribution of a processing-centered model is to ask how industrial formulation, matrix disruption, rapid eating, selected additives, processing-derived compounds, hyperpalatability, and displacement of microbial substrates may initiate or intensify these broader pathways.

### 6.1. Gut Microbiome Dysbiosis and Loss of Microbial Resilience

UPF-rich diets are generally lower in intact fiber and phytochemical diversity than minimally processed plant-forward diets. This can reduce microbial substrate availability and lower ecological resilience. Human observational evidence connects UPF consumption with altered gut microbial profiles, while preclinical and additive-focused studies suggest that specific formulation agents can alter microbial composition, mucus biology, and intestinal inflammation [14,18,19]. The evidence is strongest for the general principle that diet quality and fermentable substrates shape the microbiome; it is more variable for individual additives and UPF subtypes.

Human microbiome studies support this general direction. Studies in Spanish adults and women with differing UPF consumption have reported microbial differences according to processing exposure [74,75]. Other studies have linked UPF intake with fecal microbiota and metabolite signatures in large Mediterranean cohorts [76], and with gut microbial features in Japanese adults [77]. Broader microbiome reviews emphasize that dysbiosis should be interpreted as an ecological and functional shift rather than a single microbial signature, because host diet, sex, adiposity, socioeconomic status, medications, and geography all shape microbial communities [78,79,80]. The findings are not uniform because microbiome studies differ in sequencing approach, dietary instruments, geography, medication adjustment, and analytic pipelines. Still, they support the premise that processing level can track with microbial ecology beyond simple macronutrient intake.

### 6.2. SCFA Depletion and Intestinal Barrier Dysfunction

SCFAs, especially butyrate, support epithelial energy metabolism, tight-junction regulation, mucus layer function, and immune tolerance. Diets depleted of fermentable fiber may reduce SCFA-producing microbial taxa and weaken barrier protection. In the proposed framework, SCFA depletion is not simply a gut event: it may permit greater translocation of microbial products into portal circulation, thereby exposing the liver to inflammatory stimuli and increasing downstream systemic immune activation. Because SCFAs can also influence microglial maturation and neuroimmune function, reduced SCFA signaling may link gut dysbiosis with brain vulnerability [81,82].

This pathway is particularly relevant to UPF substitution. Replacing UPFs with whole grains, legumes, fruits, vegetables, nuts, and minimally processed plant foods increases fermentable substrates and polyphenol diversity, potentially restoring microbial metabolites that regulate barrier function and immune tone. This is one reason dietary substitution may be biologically different from calorie restriction alone.

### 6.3. Metabolic Endotoxemia and Innate Immune Activation

Metabolic endotoxemia provides one of the most coherent mechanistic bridges from UPFs to MASLD and brain aging. Dysbiosis and barrier dysfunction can increase LPS translocation into portal circulation. LPS activates Toll-like receptor 4 signaling and downstream nuclear factor-κB and inflammasome-related pathways, including NLRP3, in hepatic and immune cells. In the liver, this may promote Kupffer cell activation, hepatocellular injury, steatohepatitis, and fibrogenesis. Systemically, chronic cytokine exposure may impair endothelial function, weaken BBB integrity, and prime microglia toward exaggerated inflammatory responses [14,83].

The LPS pathway is attractive because it connects gut, liver, and brain in a directional manner: intestinal barrier injury precedes portal exposure; portal exposure activates hepatic innate immunity; hepatic inflammation contributes to systemic cytokine and vascular responses; and chronic systemic inflammation can affect BBB permeability and neuroimmune tone. However, human measurement remains difficult. LPS is labile and challenging to quantify; LPS-binding protein, soluble CD14, endotoxin-core antibodies, and inflammatory proteomics may be more practical but are imperfect proxies.

## 7. Evidence Linking UPFs to Cognitive Aging and Dementia

### 7.1. Cognitive Decline and Executive Dysfunction

The cognitive evidence is newer than the cardiometabolic evidence but increasingly coherent. In the Brazilian Longitudinal Study of Adult Health (ELSA-Brasil), 10,775 adults were followed for a median of 8 years. Participants with higher UPF consumption had faster global cognitive decline and faster executive function decline compared with those in the lowest UPF consumption group [84]. The executive function signal is mechanistically important because executive dysfunction is commonly linked to vascular injury, insulin resistance, white matter disease, and metabolic brain aging, not only to Alzheimer-type neurodegeneration.

In the REasons for Geographic and Racial Differences in Stroke study, a 10% higher relative intake of UPFs was associated with higher risk of incident cognitive impairment and stroke, while higher intake of unprocessed or minimally processed foods was associated with lower risk [85]. These associations persisted after accounting for Mediterranean, DASH, and MIND dietary pattern scores, suggesting that processing classification may capture information not fully represented by conventional diet-quality scores.

Cross-sectional and cohort studies in older adults have reported similar signals, although with the usual limitations of dietary assessment and residual confounding. NHANES analyses have linked higher UPF intake with poorer cognitive performance in older U.S. adults [86]; studies in older adults with type 2 diabetes suggest that UPF consumption may be associated with cognitive decline in a metabolically vulnerable population [87]; and recent work in nationally representative older U.S. samples has examined impairment across multiple cognitive domains [88]. A systematic review on UPFs and brain health similarly concluded that cognitive and mental-health evidence is accumulating but remains heterogeneous in outcome measurement and confounder control [89]. These studies are useful because they move beyond dementia endpoints and capture earlier phenotypes, including attention, processing speed, memory, and executive function.

### 7.2. Dementia and Dementia Subtype Outcomes

In a UK Biobank prospective cohort of 72,083 adults aged 55 years or older without dementia at baseline, each 10% increase in UPF consumption was associated with higher risk of all-cause dementia, Alzheimer disease, and vascular dementia during a median follow-up of 10 years [90]. Replacement modeling suggested that substituting 10% of UPF weight with unprocessed or minimally processed foods was associated with lower dementia risk. These findings are observational, but the replacement model is useful because it maps more directly onto dietary counseling: the clinical question is not merely whether UPFs are harmful, but which foods should replace them.

Meta-analytic and cohort evidence remains emerging. A systematic review and meta-analysis of observational studies reported that high UPF intake was associated with dementia in adults, but the authors emphasized heterogeneity and the need for stronger prospective data [91]. Framingham Heart Study analyses have investigated UPF intake in relation to dementia and Alzheimer disease risk [92]. More recent studies have also begun to examine UPF categories rather than total UPF exposure, an important advance because processed meat, sugar-sweetened beverages, sweet bakery products, and ultra-processed mixed dishes may not carry the same cognitive signal [93].

The brain-health outcome space is broader than dementia. UPF consumption has been associated with stroke in REGARDS [85], and stroke sits directly on the vascular pathway linking MASLD, endothelial dysfunction, hypertension, dyslipidemia, and cognitive aging. Other studies have extended the question to neurodegenerative disorders, prodromal Parkinson disease, brain-health risk scores, and major brain-disorder composites [94,95]. These findings should be treated as hypothesis-generating, but they reinforce the need to study processing exposure across vascular, metabolic, and neurodegenerative outcomes rather than in dementia alone.

The cognitive evidence summarized in Table 3 is less mature than the liver evidence but is moving in the same direction: away from total UPF intake alone and toward replacement models, subtype analyses, domain-specific cognition, stroke and vascular pathways, and broader neurodegenerative outcomes. This is important because a processing-centered framework predicts especially strong relevance to executive dysfunction, vascular cognitive impairment, and metabolic brain aging, even if Alzheimer-specific causality remains uncertain.

### 7.3. Interpretation Through Metabolic and Vascular Brain Aging

The most defensible interpretation is not that UPFs specifically explain Alzheimer disease. Rather, UPFs may be particularly relevant to vascular cognitive impairment, executive dysfunction, stroke, insulin-resistance-related cognitive vulnerability, and broader metabolic brain aging. This distinction matters because it aligns the cognitive evidence with the liver and gut evidence: UPFs appear most consistently linked to metabolic, inflammatory, and vascular pathways, all of which influence brain aging trajectories.

Mechanistic reviews of UPFs and neuropsychiatric outcomes point to a similar convergence: altered reward processing, gut dysbiosis, low-grade inflammation, oxidative stress, insulin resistance, BBB disruption, and changes in microbial metabolites may all contribute to cognitive and mood-related outcomes [96,97,98]. The strongest causal evidence is still missing, but the pattern is coherent enough to justify integrated liver–brain trials in high-risk metabolic populations.

### 7.4. BBB Disruption, Microglial Activation, and Neuroinflammation

The brain is sensitive to systemic inflammation and vascular dysfunction. Circulating cytokines, endothelial injury, altered lipid species, insulin resistance, microbial-derived signals, and bile acid perturbation can impair BBB function and promote microglial activation. Microglia integrate peripheral immune cues with local signals from neurons, astrocytes, and the vasculature. Chronic low-grade inflammatory input may not produce acute encephalopathy, but it may accelerate long-term cognitive vulnerability, especially in people with existing vascular risk, APOE ε4 genotype, sleep disturbance, depression, or socioeconomic adversity.

This pathway also helps explain why executive function may be a particularly sensitive outcome. Executive dysfunction is often linked to frontal–subcortical circuits, white matter integrity, and cerebral small-vessel disease. UPF-related MASLD may therefore affect cognition through vascular and neuroimmune injury even when Alzheimer-type pathology is not the dominant mechanism. Neurofilament light, glial fibrillary acidic protein, white matter hyperintensity volume, cerebral blood flow, and BBB-related imaging or fluid markers could help test this hypothesis.

### 7.5. Biological Aging and Metabolic Brain Aging

The most integrative concept is metabolic brain aging. UPFs may contribute to cognitive aging not only through single disease pathways but through cumulative biological stress: insulin resistance, oxidative stress, mitochondrial dysfunction, chronic inflammation, endothelial dysfunction, adiposity, dysregulated bile acids, and possibly epigenetic aging. MASLD may intensify this pattern because it reflects long-term failure of metabolic buffering in a central organ. This perspective shifts the question from whether UPFs are independently linked to dementia to whether UPF-rich dietary environments accelerate a multi-organ aging phenotype in which the liver and brain age together.

This concept is compatible with biological aging research. Metabolic inflammation, mitochondrial dysfunction, lipid droplet biology, cellular senescence, and disrupted autophagy are shared features of liver aging, vascular aging, and neurodegeneration [73]. A processing-centered framework encourages future studies to measure aging biomarkers alongside organ-specific endpoints rather than treating liver fat, cognition, and inflammation as disconnected outcomes.

## 8. Clinical and Public Health Implications

### 8.1. Food Environment, Equity, and Implementation

UPF consumption is shaped by price, availability, working hours, food marketing, neighborhood food environments, cooking facilities, school and workplace food provision, and social stress. A processing-centered framework should therefore avoid framing UPF intake solely as an individual failure. Public health strategies may include front-of-package labeling, restrictions on marketing to children, procurement policies that improve minimally processed food availability in schools, hospitals, and workplaces, taxation or reformulation standards targeting sugar-sweetened beverages and highly processed products, and subsidies or access programs for minimally processed foods.

Equity is central because both UPF exposure and MASLD risk are socially patterned. Food insecurity has been associated with MASLD prevalence and liver-related mortality [99], and global food-system structures may shape MASLD risk through development-stratified pathways [100]. Public health approaches should therefore pair UPF reduction with affordability, access, culinary infrastructure, realistic preparation time, and culturally appropriate minimally processed foods. The goal is not to moralize convenience, but to make lower-UPF substitutions easier, affordable, and socially feasible.

### 8.2. MASLD Patients as a Cognitive Prevention Target Group

People with MASLD may represent a high-risk group for cognitive prevention, particularly when MASLD coexists with obesity, type 2 diabetes, hypertension, sleep disturbance, depression, or low physical activity. Liver-focused dietary counseling should therefore be integrated with cardiometabolic and cognitive health goals. In practice, this means monitoring not only liver enzymes and fibrosis risk, but also vascular risk factors, sleep, mood, physical activity, and cognitive complaints when clinically relevant.

This does not mean all MASLD patients require formal neuropsychological testing. Rather, MASLD can be used as a clinical signal of systemic metabolic vulnerability. Patients with advanced fibrosis, diabetes, recurrent depression, sleep apnea, or vascular disease may be especially relevant for cognitive prevention strategies. Conversely, cognitive symptoms can undermine adherence to complex lifestyle recommendations, making simplified dietary substitution and food environment support more useful than highly demanding dietary prescriptions.

### 8.3. Dietary Substitution, Not Only Calorie Restriction

Clinical counseling should not reduce UPF guidance to calorie restriction alone. The more actionable message is substitution: replace sugar-sweetened beverages with water or unsweetened beverages; replace refined packaged snacks with fruit, nuts, legumes, or minimally processed whole-grain foods; replace processed meats with legumes, fish, poultry, or other minimally processed protein sources; and replace ready-to-eat industrial meals with simple plant-forward meals when feasible. This substitution frame is supported by dementia replacement modeling [90] and is more realistic than telling patients only to “eat less”.

Substitution also fits MASLD guidance, which emphasizes weight loss when appropriate but increasingly recognizes diet quality, cardiometabolic risk reduction, and long-term adherence as central goals [2,3]. A minimally processed, plant-forward approach can simultaneously reduce energy density, improve fiber and polyphenol intake, support microbial substrate availability, and reduce repeated exposure to sugar-sweetened beverages, processed meats, and highly refined snacks. For clinical care, the most practical first step may be identifying the largest UPF sources in a patient’s diet and replacing one or two categories at a time.

## 9. Challenges and Limitations

Several limitations should temper interpretation. First, NOVA classification is heterogeneous, and risk may differ markedly across UPF subtypes. Second, residual confounding remains a major concern because UPF intake is socially patterned and correlated with multiple health behaviors. Third, reverse causation is possible: people with early cognitive change, depression, disability, or time scarcity may rely more heavily on convenient foods. Fourth, dietary assessment error is substantial because FFQs and 24 h recalls may lack the ingredient, brand, and preparation information required for precise UPF classification. Fifth, MASLD outcome measurement varies widely across studies, from fatty liver index and liver enzymes to elastography, MRI, administrative codes, and biopsy. Sixth, the evidence base lacks integrated trials that test whether reducing UPFs improves MASLD and cognitive outcomes through measurable gut, inflammatory, vascular, and bile acid pathways.

The current evidence therefore supports UPF consumption as a plausible and increasingly supported risk marker and exposure, but causal pathways remain incompletely established. The field needs stronger exposure assessment, better subtype resolution, and intervention studies designed around mechanistic endpoints.

There are also conceptual controversies. Critics argue that NOVA may sometimes classify foods with favorable nutrient profiles as UPFs while classifying some energy-dense homemade foods as less processed. Supporters argue that processing captures food structure, formulation, and commercial design features missed by nutrient profiling. Both arguments are valid. The best path forward is not to abandon processing classification but to improve it through subtype analyses, nutrient-processing joint models, additive mapping, and replacement designs.

Finally, MASLD itself is heterogeneous. Steatosis, steatohepatitis, fibrosis, and cardiometabolic comorbidity may carry different implications for brain aging. Studies relying on fatty liver index or administrative codes may not identify the same biological phenotype as imaging- or elastography-based studies. This heterogeneity may explain why some MASLD-dementia studies are null or inverse while fibrosis-brain studies appear more consistent [54,56,65].

## 10. Future Directions

**Figure 3:**
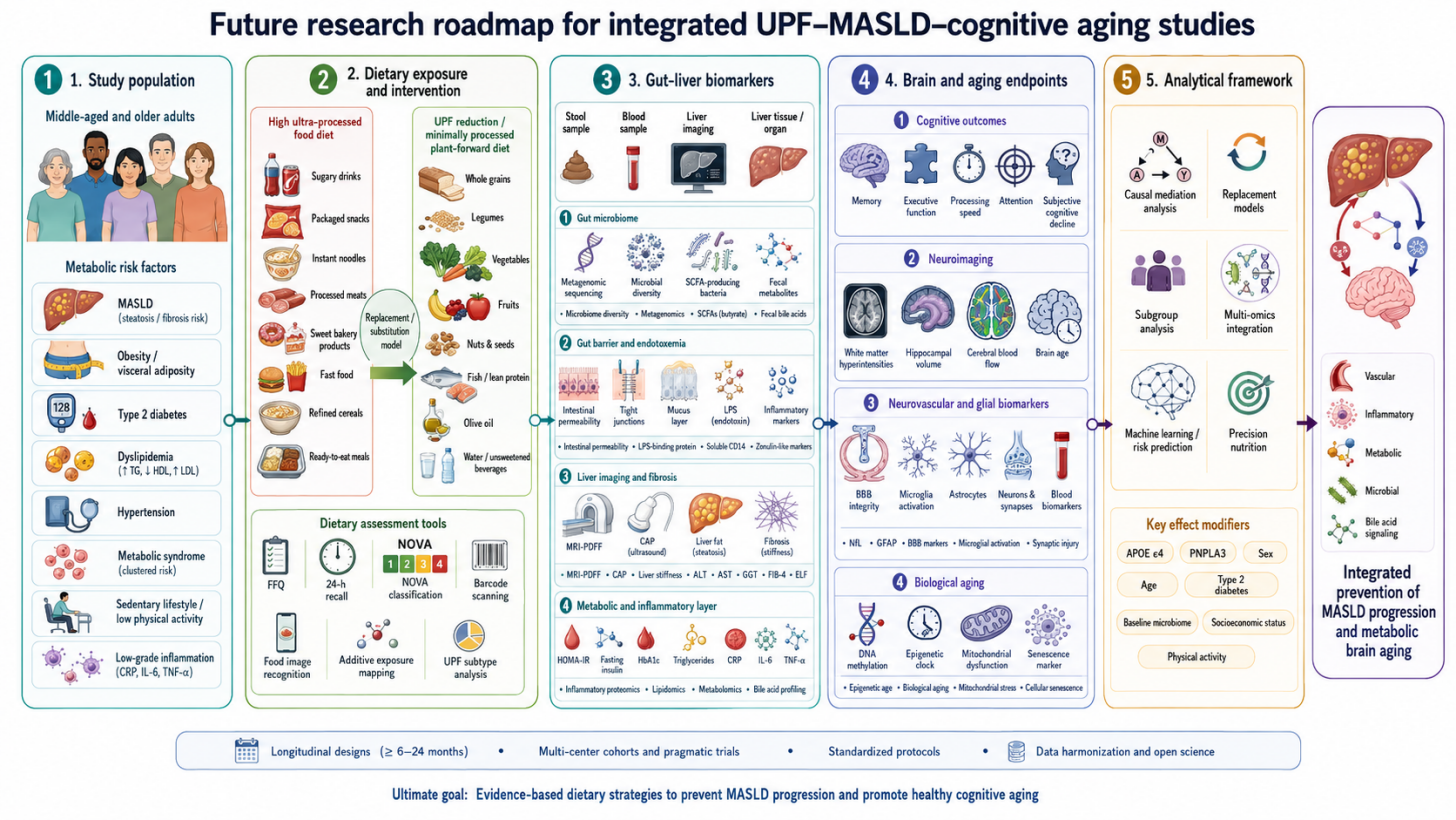
Future research roadmap for integrated ultra-processed food (UPF)-metabolic dysfunction-associated steatotic liver disease (MASLD)-cognitive aging studies. Priority designs should combine careful UPF exposure assessment, UPF-reduction or minimally processed food substitution interventions, liver imaging and fibrosis markers, gut microbiome and metabolite profiling, inflammatory and vascular biomarkers, neuroimaging, cognitive testing, biological aging measures, and analytical strategies including causal mediation, replacement models, subgroup analyses, multi-omics integration, risk prediction, and precision nutrition. The figure was assembled in Microsoft PowerPoint and exported as PNG.

Future research should be designed around the full axis rather than around isolated pairwise associations. Figure 3 outlines a research roadmap integrating study population, dietary exposure and intervention, gut–liver biomarkers, brain and aging endpoints, and analytical frameworks.

### 10.1. UPF-Reduction Trials with Liver and Cognitive Endpoints

An ideal trial would enroll middle-aged or older adults with MASLD and metabolic risk. The intervention would reduce UPF intake through substitution with minimally processed, plant-forward foods; the comparator would receive standard healthy-eating advice. Follow-up should last at least 6–24 months. Liver outcomes should include MRI-proton density fat fraction, controlled attenuation parameter, ALT, AST, FIB-4, and, where feasible, enhanced liver fibrosis score. Cognitive outcomes should include executive function, memory, processing speed, subjective cognitive decline, and, in longer studies, incident mild cognitive impairment. Mechanistic endpoints should include gut microbiome composition and function, fecal or plasma SCFAs, bile acid profiling, LPS-binding protein, CRP, IL-6, TNF-α, metabolomics, lipidomics, and inflammatory proteomics. Optional neuroimaging could assess white matter hyperintensity burden, hippocampal volume, cerebral blood flow, and BBB-related markers. Candidate endpoint domains are summarized in Table 4.

Trial design should distinguish between UPF reduction and general healthy-eating advice. A low-UPF intervention should document changes in NOVA groups, UPF subtypes, dietary fiber, added sugars, saturated fat, sodium, energy density, eating rate, and dietary cost. Without these measures, it will be impossible to know whether benefits arise from reduced processing, improved nutrient profile, weight loss, increased plant foods, or their combination. Pragmatic trials in real-world clinics may be complemented by shorter controlled feeding studies designed to isolate food structure, additive exposure, and eating-rate mechanisms.

### 10.2. Multi-Omics and Biomarker Integration

Future studies should combine metagenomics, metabolomics, lipidomics, bile acid profiling, inflammatory proteomics, and biological aging markers. The UK Biobank omics analysis of UPF signatures and liver outcomes illustrates the value of moving beyond self-reported diet and single biomarkers [35]. Similar omics approaches should be linked to cognitive testing and neuroimaging.

Multi-omics studies should prioritize interpretability. Rather than reporting large biomarker panels without a causal model, studies should pre-specify pathways: microbial substrate depletion and SCFA signaling; endotoxemia and innate immune activation; bile acid-FXR-TGR5 signaling; hepatic lipotoxicity and inflammatory proteins; vascular dysfunction; and neuroinflammation. This would make omics evidence more actionable for intervention design.

### 10.3. Causal Mediation and Replacement Models

Cohort studies should routinely test UPF subtype associations, replacement models, mediation by MASLD markers or gut biomarkers, and effect modification by APOE ε4, PNPLA3, type 2 diabetes, sex, age, socioeconomic status, and baseline diet quality. Mediation analyses should be interpreted cautiously, but they can help prioritize pathways for trials.

Replacement models are especially important because they answer a clinically meaningful question: what happens when a proportion of UPFs is replaced by minimally processed foods, culinary ingredients, or processed but not ultra-processed foods? Mediation models should then ask whether changes in liver fat, fibrosis markers, inflammatory proteins, bile acids, or gut microbial metabolites explain part of the UPF-cognition association.

### 10.4. Better Exposure Assessment

Dietary exposure assessment must evolve beyond conventional FFQs. Barcode scanning, food image recognition, commercial food composition databases, ingredient-level additive mapping, and repeated short dietary recalls could improve UPF classification. These tools would also help distinguish processing level from nutrient profile, food category, and socioeconomic context.

Future datasets should also capture food form and context: liquid versus solid calories, ready-to-eat versus home-prepared foods, eating speed, meal timing, packaging, additive classes, price, and availability. These features are not peripheral. They are part of what makes UPFs processing-related exposures rather than merely collections of nutrients.

## 11. Conclusions

UPFs should be conceptualized not merely as nutrient-poor foods but as processing-related dietary exposures that may perturb the gut–liver–brain axis. Current evidence links higher UPF consumption with MASLD/NAFLD-related outcomes and with cognitive decline, cognitive impairment, stroke, and dementia-related outcomes, although causality remains incompletely established. Mechanistically, UPFs may disrupt the food matrix, reduce microbial substrates, impair intestinal barrier integrity, promote metabolic endotoxemia, alter bile acid signaling, and amplify hepatic and systemic inflammation. MASLD may serve as a hepatic metabolic amplifier that converts processing-related dietary stress into systemic inflammatory, vascular, microbial, and bile acid signals affecting brain aging. The next generation of studies should test UPF substitution using integrated liver, gut, inflammatory, vascular, omics, neuroimaging, and cognitive endpoints. The key messages and remaining questions are summarized in Box 1 and Box 2, respectively.

Box 1 Key messages.

- UPFs should be conceptualized as processing-related exposures, not merely as nutrient-poor foods.
- MASLD may amplify UPF-related gut-derived and metabolic signals into systemic inflammatory and vascular insults.
- Cognitive outcomes most plausibly affected include executive dysfunction, vascular cognitive impairment, stroke-related pathways, and metabolic brain aging.
- Future trials should test UPF substitution using integrated liver, gut, inflammatory, vascular, omics, and cognitive endpoints.

Box 2 **Outstanding questions.**

- Which UPF subtypes are most strongly linked to MASLD and cognitive aging?
- Does MASLD mediate or modify the UPF-cognition association?
- Are liver, gut, inflammatory, and cognitive effects reversible after UPF reduction?
- Which biomarkers best capture processing-related gut–liver–brain dysfunction?
- Do APOE ε4, PNPLA3, type 2 diabetes, sex, age, and socioeconomic status modify effects?

## Figures and Tables

**Table 1:** Processing specificity of candidate pathways linking ultra-processed foods with gut–liver–brain outcomes.

Pathway Category	Examples	Processing Specificity	Gut and Liver Relevance	Brain and Cognitive Relevance
Processing-specific or processing-related	Matrix disruption, rapid eating, hyperpalatability, selected additives, processing-derived compounds	Highest; these mechanisms are most directly linked to industrial formulation and food structure	Altered satiety and digestion, dysbiosis, mucus layer or barrier stress, oxidative and inflammatory signaling	Neuroimmune and vascular vulnerability through gut-derived and systemic signals
Diet-quality-related	Low fiber, low polyphenol diversity, refined starches, added sugars, high sodium	Moderate; these pathways may reflect both UPF exposure and poor dietary quality	Reduced microbial substrates, lower SCFA production, hepatic de novo lipogenesis, hypertriglyceridemia	Insulin resistance, endothelial dysfunction, vascular cognitive impairment
Subtype-specific	Sugar-sweetened beverages, processed meats, sweet bakery products, savory snacks, ready-to-eat meals	Moderate; biological relevance varies by UPF subtype rather than total UPF exposure alone	Fructose-related lipogenesis, nitrites, oxidized lipids, advanced glycation end products, energy density	Stroke, small-vessel injury, metabolic brain aging, domain-specific cognitive decline
Adiposity and cardiometabolic mediation	Visceral adiposity, insulin resistance, dyslipidemia, hypertension, systemic inflammation	Lower; not unique to UPFs but may mediate or amplify UPF-associated risk	Hepatic lipid flux, lipotoxicity, Kupffer cell activation, fibrosis progression	BBB dysfunction, microglial priming, executive dysfunction, dementia vulnerability

**Table 2:** Selected evidence linking UPF consumption with MASLD/NAFLD-related outcomes.

Study	Design and Population	Exposure Assessment	Outcome Definition and Terminology	Main Finding and Main Limitation
Henney et al. [32]	Systematic review and meta-analysis; adult observational studies	NOVA-based UPF intake	Study-defined NAFLD	High versus low UPF intake associated with higher NAFLD risk; heterogeneous designs and diagnostic methods
Grinshpan et al. [31]	Systematic review	UPF intake by study-specific dietary instruments	NAFLD, metabolic syndrome, insulin resistance	Synthesized convergence between UPF exposure and metabolic liver risk; limited longitudinal and mechanistic evidence
Zhang et al. [33]	Updated dose-response meta-analysis; 10 articles	Study-specific UPF estimates	NAFLD	Highest UPF intake associated with 22% higher NAFLD risk; substantial between-study heterogeneity
Zhao et al. [34]	UK Biobank prospective cohort; 173,889 adults	Repeated 24 h recalls classified by NOVA	NAFLD, fibrosis/cirrhosis, severe liver disease	Higher UPF intake associated with liver outcomes; dietary recalls may misclassify processing level
Zhang et al. [43]	UK Biobank prospective cohort; 143,073 adults	UPF intake from dietary assessment	Severe NAFLD	Extended prospective evidence to severe outcomes; definition based on available cohort data
Zhao et al. [35]	UK Biobank omics cohort	UPF intake plus metabolomic/proteomic signatures	MASLD and adverse liver outcomes	UPF-related omics signatures associated with MASLD and inflammation; observational omics cannot prove causality
Zhang et al. [41]	Tianjin Chronic Low-grade Systemic Inflammation and Health cohort	UPF intake and selected subtypes	Incident NAFLD	Prospective Asian cohort evidence; food subtypes and confounding may differ by culture
Konieczna et al. [39]	PREDIMED-Plus older adults with overweight/obesity and metabolic syndrome	UPF intake	NAFLD-related biomarkers	Supported relevance in a high-risk Mediterranean cohort; older high-risk sample limits generalizability
Zhao et al. [37]	U.S. national survey; adolescents and adults	UPF intake from dietary recalls	Odds of NAFLD	Positive associations in adolescents and adults; cross-sectional design
Liu et al. [38]	NHANES 2011–2018 population analysis	UPF intake from survey dietary data	NAFLD risk	Added U.S. population-based evidence; residual confounding and dietary recall error
Song et al. [36]	NHANES 2017–2020 cross-sectional analysis	24 h recalls classified by NOVA	CAP-defined steatosis and liver stiffness	Higher UPF intake associated with steatosis; fibrosis signal less consistent
Fu et al. [42]	Korean Genome and Epidemiology Study prospective analysis	UPF intake and subtypes	Incident NAFLD	Demonstrated East Asian prospective evidence; NOVA mapping may vary by food culture
Lee et al. [44] and Buytaert et al. [45]	Pediatric/adolescent obesity and NHANES analyses	UPF intake	Metabolic disorders and adolescent MASLD	Support early-life relevance; pediatric results should not be directly extrapolated to adults
Geladari et al. [46] and Moslehi et al. [47]	Recent review and Tehran Lipid and Glucose Study	UPF or processing-degree exposure	MASLD evidence	Broadened regional and MASLD-terminology evidence; newer MASLD definitions remain unevenly applied

**Table 3:** Selected evidence linking UPF consumption with cognitive aging and brain health outcomes.

Study	Design and Population	Exposure Assessment	Outcome Definition	Main Finding and Main Limitation
Gomes Goncalves et al. [84]	ELSA-Brasil prospective cohort; 10,775 adults	UPF intake by dietary assessment and NOVA classification	Global cognition and executive function change	Higher UPF intake associated with faster decline; residual confounding remains possible
Li et al. [90]	UK Biobank prospective cohort; 72,083 adults aged 55 years or older	UPF weight percentage and replacement models	All-cause dementia, Alzheimer disease, vascular dementia	UPF increment associated with dementia risk and substitution with minimally processed foods associated with lower risk; observational design
Bhave et al. [85]	REGARDS prospective cohort	Relative UPF intake	Incident cognitive impairment and stroke	Associations persisted after diet-quality scores; processing classification may still be confounded by lifestyle factors
Henney et al. [91]	Systematic review and meta-analysis	Study-specific UPF estimates	Dementia in adults	High UPF intake associated with dementia; evidence remains heterogeneous and observational
Weinstein et al. [92]	Framingham Heart Study	UPF consumption	Dementia and Alzheimer disease	Community-based prospective evidence; requires replication across diverse cohorts
Cardoso et al. [86]	NHANES 2011–2014 older adults	UPF intake from recalls	Cognitive performance	Higher UPF intake associated with poorer performance; cross-sectional design and reverse causation possible
Weinstein et al. [87]	Older adults with type 2 diabetes	UPF consumption	Cognitive decline	Signal in a metabolically vulnerable population; diabetes-specific context limits generalization
Seago et al. [93]	Longitudinal panel study of middle-aged and older adults	UPF categories and total intake	Incident cognitive impairment	UPF categories showed differential associations; highlights subtype heterogeneity
Lee et al. [88]	Nationally representative older U.S. adults	UPF intake	Multiple cognitive domains	Examined domain-specific impairment; newer evidence needs longitudinal confirmation
Claudino et al. [89]	Systematic review	UPF consumption	Alzheimer disease and brain-health outcomes	Synthesized emerging evidence; outcome definitions and covariate control vary
Pourmotabbed et al. [94]	Systematic review and dose-response meta-analysis of large-scale cohorts	UPF intake	Neurodegenerative disorders	Extended the question beyond dementia alone; broad outcome grouping may dilute mechanisms
Maffetone and Laursen [95] and Grant et al. [96]	Public health and mechanistic reviews	Refined carbohydrate or dietary-pattern evidence	Brain health and neuropsychiatric pathways	Support mechanistic plausibility; mostly inferential rather than trial evidence
Wang et al. [53]	Systematic review and meta-analysis	NAFLD exposure rather than UPF exposure	Cognitive impairment and dementia	Connects liver–brain pathway; not direct UPF evidence

**Table 4:** Candidate endpoints for integrated UPF-MASLD-cognitive aging studies.

Domain	Candidate Biomarkers or Endpoints
Dietary exposure	NOVA-classified UPF energy or weight percentage; UPF subtype intake; replacement models; barcode or image-assisted food records; additive exposure mapping
Gut	Microbiome diversity and metagenomic function; SCFAs; fecal bile acids; zonulin or other barrier markers; LPS-binding protein
Liver	MRI-PDFF; controlled attenuation parameter; liver stiffness; ALT; AST; GGT; FIB-4; enhanced liver fibrosis score
Metabolic	HOMA-IR; fasting insulin; HbA1c; triglycerides; LDL-C; HDL-C; blood pressure; visceral adiposity
Inflammation	CRP; IL-6; TNF-α; monocyte activation markers; NLRP3-related markers; inflammatory proteomics
Brain and cognition	Executive function; processing speed; episodic memory; subjective cognitive decline; white matter hyperintensities; hippocampal volume; cerebral blood flow; neurofilament light; GFAP

## Data Availability

No new data were created or analyzed in this study. Data sharing is not applicable to this article.

## Supplementary Materials

The following supporting information can be downloaded at: https://www.mdpi.com/article/10.3390/nu18132041/s1, Table S1: Search strategy and selection criteria.

## Abbreviations

The following abbreviations are used in this manuscript:

BBB	Blood–brain barrier
CAP	Controlled attenuation parameter
FFQ	Food frequency questionnaire
FXR	Farnesoid X receptor
GFAP	Glial fibrillary acidic protein
LPS	Lipopolysaccharide
MASLD	Metabolic dysfunction-associated steatotic liver disease
MASH	Metabolic dysfunction-associated steatohepatitis
MRI-PDFF	Magnetic resonance imaging-proton density fat fraction
NAFLD	Non-alcoholic fatty liver disease
NLRP3	NOD-like receptor family pyrin domain-containing 3
SCFA	Short-chain fatty acid
TGR5	Takeda G protein-coupled receptor 5
UPF	Ultra-processed food
