# Supplementary material for: Ultra-Processed Foods, MASLD, and Cognitive Aging: A Processing-Centered Gut–Liver–Brain Axis Perspective
Source: Nutrients. 2026 Jun 23;18(13):2041. doi: 10.3390/nu18132041 (PMC13363463; doi:10.3390/nu18132041)
Supplement: Supplementary file 1 [file nutrients-18-02041-s001.zip › nutrients-4346039-supplementary.pdf]

Supplementary Table S1. Search Strategy and Selection Criteria

This supplementary table reports the structured search strategy used for the narrative review entitled "Ultra-Processed Foods, MASLD, and Cognitive Aging: A Processing-Centered Gut-Liver-Brain Axis Perspective". The PubMed/MEDLINE strings were adapted for Web of Science Core Collection and Scopus by removing field tags and using equivalent title, abstract, and keyword fields where appropriate. Searches covered January 2010 to 11 May 2026. Earlier landmark mechanistic studies were included when necessary to define core concepts.

Search modules

```
("ultra-processed food*"[Title/Abstract] OR "ultraprocessed food*"[Title/Abstract] OR "ultra
    processed food*"[Title/Abstract] OR "NOVA classification"[Title/Abstract] OR "food processing"[
    Title/Abstract])

AND

("metabolic dysfunction-associated steatotic liver disease"[Title/Abstract] OR MASLD[Title/Abstract
    ] OR "metabolic dysfunction-associated steatohepatitis"[Title/Abstract] OR MASH[Title/Abstract]
     OR "non-alcoholic fatty liver disease"[Title/Abstract] OR "nonalcoholic fatty liver disease"[
    Title/Abstract] OR NAFLD[Title/Abstract] OR "hepatic steatosis"[Title/Abstract] OR "fatty liver
    "[Title/Abstract])
```

```
("ultra-processed food*"[Title/Abstract] OR "ultraprocessed food*"[Title/Abstract] OR "ultra
    processed food*"[Title/Abstract] OR "NOVA classification"[Title/Abstract] OR "food processing"[
    Title/Abstract])

AND

("cognitive decline"[Title/Abstract] OR "cognitive aging"[Title/Abstract] OR "cognitive ageing"[
    Title/Abstract] OR cognition[Title/Abstract] OR "executive function"[Title/Abstract] OR
    dementia[Title/Abstract] OR Alzheimer*[Title/Abstract] OR "mild cognitive impairment"[Title/
    Abstract] OR "brain aging"[Title/Abstract] OR "brain ageing"[Title/Abstract])
```

```
("ultra-processed food*"[Title/Abstract] OR "ultraprocessed food*"[Title/Abstract] OR "ultra
    processed food*"[Title/Abstract] OR "NOVA classification"[Title/Abstract])

AND

("gut microbiome"[Title/Abstract] OR "gut microbiota"[Title/Abstract] OR "intestinal permeability"[
    Title/Abstract] OR "leaky gut"[Title/Abstract] OR endotoxemia[Title/Abstract] OR
    lipopolysaccharide[Title/Abstract] OR LPS[Title/Abstract] OR "short-chain fatty acid*"[Title/
    Abstract] OR SCFA[Title/Abstract] OR "bile acid*"[Title/Abstract] OR FXR[Title/Abstract] OR
    TGR5[Title/Abstract] OR "blood-brain barrier"[Title/Abstract] OR neuroinflammation[Title/
    Abstract] OR microglia[Title/Abstract] OR "insulin resistance"[Title/Abstract])
```

```
("metabolic dysfunction-associated steatotic liver disease"[Title/Abstract] OR MASLD[Title/Abstract
    ] OR "non-alcoholic fatty liver disease"[Title/Abstract] OR "nonalcoholic fatty liver disease"[
    Title/Abstract] OR NAFLD[Title/Abstract])
```

```
AND

("cognitive decline"[Title/Abstract] OR "cognitive aging"[Title/Abstract] OR dementia[Title/
    Abstract] OR Alzheimer*[Title/Abstract] OR "mild cognitive impairment"[Title/Abstract] OR
    neuroinflammation[Title/Abstract] OR "brain aging"[Title/Abstract])
```

Web of Science Core Collection search strategy

Database: Web of Science Core Collection. Time span: 1 January 2010 to 11 May 2026. Document types: Article; Review; Early Access. Language: English.

```
TS=(("ultra-processed food*" OR "ultraprocessed food*" OR "ultra processed food*" OR "NOVA
    classification" OR "food processing" OR "industrial food*" OR "processed food*") AND (MASLD OR
    "metabolic dysfunction-associated steatotic liver disease" OR NAFLD OR "non-alcoholic fatty
    liver disease" OR "nonalcoholic fatty liver disease" OR "hepatic steatosis" OR "liver fat" OR
    steatohepatitis OR "liver fibrosis"))
```

```
TS=(("ultra-processed food*" OR "ultraprocessed food*" OR "ultra processed food*" OR "NOVA
    classification" OR "food processing") AND ("cognitive aging" OR "cognitive decline" OR "
    cognitive impairment" OR dementia OR "Alzheimer disease" OR "vascular dementia" OR stroke OR "
    executive function" OR neurodegeneration OR "brain aging"))
```

```
TS=(("ultra-processed food*" OR "ultraprocessed food*" OR "ultra processed food*" OR "NOVA
    classification" OR "food processing" OR additive* OR emulsifier* OR sweetener*) AND ("gut
    microbiome" OR "gut microbiota" OR dysbiosis OR "intestinal permeability" OR "intestinal
    barrier" OR "leaky gut" OR lipopolysaccharide OR LPS OR "short-chain fatty acid*" OR SCFA OR "
    bile acid*" OR FXR OR TGR5 OR "blood-brain barrier" OR BBB OR neuroinflammation OR microglia OR
     endotoxemia))
```

```
TS=((MASLD OR "metabolic dysfunction-associated steatotic liver disease" OR NAFLD OR "non-alcoholic
     fatty liver disease" OR "nonalcoholic fatty liver disease" OR "hepatic steatosis" OR "liver
    fibrosis") AND ("cognitive aging" OR "cognitive decline" OR "cognitive impairment" OR dementia
    OR "Alzheimer disease" OR "vascular dementia" OR "brain structure" OR neuroimaging OR "brain
    aging" OR "blood-brain barrier" OR neuroinflammation))
```

Selection criteria

Criterion	Description
Databases	PubMed/MEDLINE, Web of Science Core Collection, and Scopus.
Time frame	January 2010 to 11 May 2026; earlier landmark mechanistic studies included selectively.
Language	English-language full-text articles were prioritized.
Included evidence	Prospective cohort studies, cross-sectional studies, randomized or controlled feeding trials, systematic reviews, meta-analyses, mechanistic human studies, and selected preclinical studies with clear relevance to human gut-liver-brain pathways.
Excluded or deprioritized evidence	Pediatric-only studies, conference abstracts without full text, studies focused only on isolated nutrients without UPF or food-processing classification, and studies centered on overt cirrhosis or hepatic encephalopathy rather than MASLD-related cognitive aging.
Review type	Structured narrative review with transparent search strategy; no duplicate screening, formal risk-of-bias scoring, or PRISMA flow diagram was performed.
